# Three-dimensional surface models of autopsied human brains constructed from multiple photographs by photogrammetry

**DOI:** 10.1371/journal.pone.0219619

**Published:** 2019-07-10

**Authors:** Hiroshi Shintaku, Mari Yamaguchi, Shuta Toru, Masanobu Kitagawa, Katsuiku Hirokawa, Takanori Yokota, Toshiki Uchihara

**Affiliations:** 1 Laboratory of Structural Neuropathology, Tokyo Metropolitan Institute of Medical Science, Setagaya-ku, Tokyo, Japan; 2 Department of Neurology and Neurological Science, Tokyo Medical and Dental University, Bunkyo-ku, Tokyo, Japan; 3 Department of Pathology, Nitobe-Memorial Nakano General Hospital, Nakano-ku, Tokyo, Japan; 4 MediaTechnology Laboratory, Tokyo Metropolitan Institute of Medical Science, Setagaya-ku, Tokyo, Japan; 5 Department of Neurology, Nitobe-Memorial Nakano General Hospital, Nakano-ku, Tokyo, Japan; 6 Department of Comprehensive Pathology, Tokyo Medical and Dental University, Bunkyo-ku, Tokyo, Japan; 7 Neuromorphomics Laboratory, Nitobe-Memorial Nakano General Hospital, Nakano-ku, Tokyo, Japan; Institut du cerveau et de la moelle epiniere, FRANCE

## Abstract

Virtual three-dimensional (3D) surface models of autopsied human brain hemispheres were constructed by integrating multiple two-dimensional (2D) photographs. To avoid gravity-dependent deformity, formalin-fixed hemispheres were placed on non-refractile, transparent acrylic plates, which allowed us to take 2D photographs from various different angles. Photogrammetric calculations using software (ReCap Pro cloud service, Autodesk, San Rafael, CA, USA) allowed us calculate the 3D surface of each brain hemisphere. Virtual brain models could be moved and rotated freely to allow smooth, seamless views from different angles and different magnifications. When viewing rotating 3D models on 2D screens, 3D aspects of the models were enhanced using motion parallax. Comparison of different brains using this method allowed us to identify disease-specific patterns of macroscopic atrophy, that were not apparent in conventional 2D photographs. For example, we observed frontal lobe atrophy in a progressive supranuclear palsy brain, and even more subtle atrophy in the superior temporal gyrus in amyotrophic lateral sclerosis-frontotemporal lobar degeneration. Thus, our method facilities recognition of gyral atrophy. In addition, it provides a much more powerful and suitable way of visualizing the overall appearance of the brain as a three-dimensional structure. Comparison of normal and diseased brains will allow us to associate different macroscopic changes in the brain to clinical manifestations of various diseases.

## Introduction

Macroscopic findings from autopsied human brains are pivotal elements that may bridge clinical manifestations to microscopic evaluation. In clinical settings, imaging techniques such as computed tomography (CT) and magnetic resonance imaging (MRI) generate three-dimensional (3D) imaging data. However, recording of autopsied brains is still limited to two-dimensional (2D) photographs, and human neuroanatomy and neuropathology lag behind clinical imaging in terms of 3-dimensionalization. Attempts to reconstruct the structures of autopsied brains and neurons into 3D constructs have been conducted previously [1, 2, 3, 4, 5]. However, there are no reports of successful reconstruction of 3D surface models that can be observed from all directions to allow in-depth viewing and recording of gyral surfaces. The aim of this study is to create 3D surface models of autopsied brains so that macroscopic and histological findings from autopsied brains can be more accurately associated with clinical manifestations.

## Materials and methods

This study was approved by the research ethics committee of Tokyo Metropolitan Institute of Medical Science (Permission number: 16–25). Three autopsied brain hemispheres from two males and one female who were either pathologically normal or diagnosed with amyotrophic lateral sclerosis (ALS) or progressive supranuclear palsy (PSP) were formalin fixed. Participants were 46–77 years old with a disease duration of 2–7 years. In all cases, according to the guidelines of the hospital, written informed concent to perform autopsy and subsequent research, was obtained from responsible family members.

### 2D-image acquisition for 3D reconstruction

**Fig 1:**
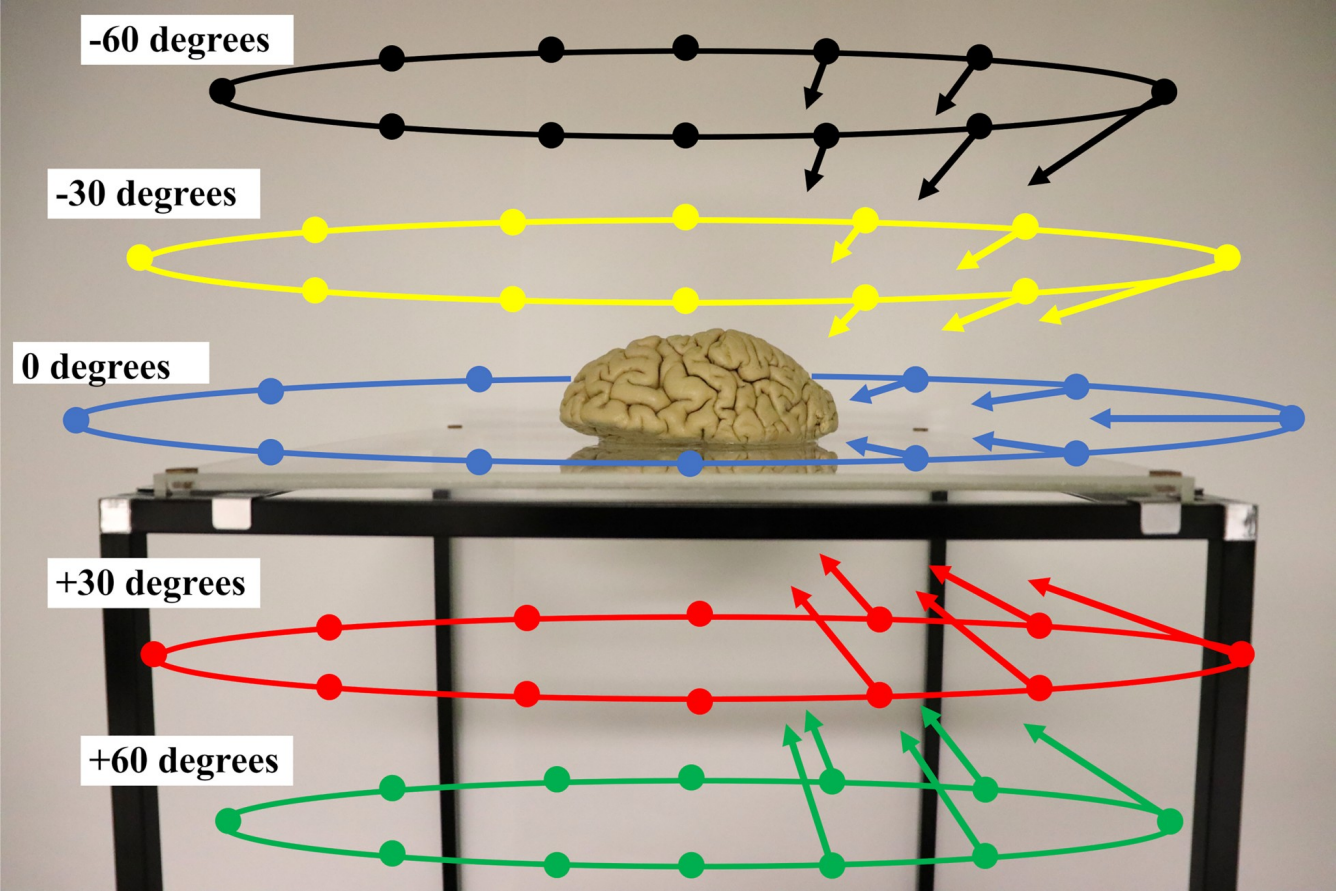
Photographing brain hemispheres. Brain hemispheres were placed at the center of a non-refractile, transparent acrylic plate on a thin frame. A thin frame supported the acrylic plate, allowing photographs to be taken from various positions. Photographs were taken at horizontal intervals of 30 degrees on the horizontal plane (blue circle with shooting points). Photographs from above (yellow circle: -30 degrees and black circle: -60 degrees) and photographs from below (red circle: +30 degrees and green circle: +60 degrees) were taken similarly at horizontal intervals of 30 degrees. Camera directions are indicated by arrows (for the right half of the figure).

Multiple photographs under similar conditions (illumination, focusing, resolution, etc.) are necessary for 3D reconstruction of brain hemispheres. However, if we use a fixed camera and illumination system, it becomes necessary to rotate the target brain hemisphere. This is not optimal since the brain hemisphere deforms due to gravity in different ways at different positions, preventing accurate 3D reconstruction. Therefore, brain hemispheres were placed on a transparent acrylic plate with minimal light reflection on a thin framework of aluminum (Fig 1). This allowed us to obtain multiple photographs from different positions (including positions below the acrylic plate) using a hand-held camera (D500, Nikon, Tokyo, Japan) and lens (AF-S Micro NIKKOR 60mm f/2.8G ED, Nikon, Tokyo, Japan). An illumination apparatus (Wireless Speedlight Commander SU-800 and Wireless Remote Speedlight SB-R200, Nikon, Tokyo, Japan) was attached to the lens so that lighting was standardized throughout the photo sessions. The color tone and shadowing of each image were manually adjusted to minimize differences. For 3D reconstruction, approximately 70% or greater overlap between neighboring pairs of images was necessary. To allow this, twelve images of horizontal projections along the acrylic plate at a horizontal interval of 30 degrees around the hemisphere were taken (Fig 1). Images from above (-60 degrees: black, -30 degrees: yellow), and images from below (+60 degrees: green, +30 degrees: red) were obtained similarly to horizontal images (0 degrees: blue) at intervals of 30 degrees (Fig 1). Additional images to highlight atrophic areas or complex convolutions were also obtained. Because images were obtained using a hand-held camera, projection angles were approximate, and the distance between the camera and the brain hemisphere was not constant.

### Image processing for 3D reconstruction

In order to construct a 3D model from 2D images, we used a method known as photogrammetry [6]. This technology has not been used much in medical studies, but is well established in various fields such as topographic map drawing and creation of building models using aerial photographs [1, 2, 7, 8, 9, 10, 11]. Usually, strict image acquisition at the same working distance (the same magnification of the object) and illumination from predefined camera positions is necessary to reconstruct the 3D surface of a target. In the present study, however, the camera position was not fixed and obtained 2D images were of slightly different magnifications. When comparing the overlapping images, it is necessary to analyze their similarities and differences since observed similarities represent target identity and observed differences provide clues to 3D representation as described below.

#### i) Identification of similarities between photographs to pinpoint common feature points

In order to transform multiple 2D photographs into a single 3D reconstruct, it is necessary to obtain reference points on the 3D surface that are shared but differently represented on multiple photographs. Although visual comparison makes it possible to identify common feature as a guide to obtain reference points, automatic identification of common feature points on overlapping photographs is challenging, especially when the magnification of the object in the photos is not identical. To identify consistent reference points (Similarity detection), original color images were first reduced to gray scale to calculate regional gradients of the intensity [12]. Next, images were blurred in stages at different scales using a Gaussian function [12]. Following this, several images were created by calculating the intensity differences between blurred images (Difference of Gaussian (DoG) images) [12]. In DoG images, points where the intensity difference between the point and the surrounding eight directions reaches an extreme value were identified [12]. If these points maintain their intensity difference values compared to surrounding points in DoG images of different scales, they can be said to be invariant to scale [12]. Intensity gradient information of areas surrounding these points were measured and used to identify feature points [12]. This method of calculation is called a Scale-Invariant Feature Transform (SIFT) algorithm [12]. Finally, feature points between images were matched based on the distribution of intensity changes, and common points were recognized between the photographs [13]. Because a sufficient number of common feature points were necessary for subsequent analysis, 70% or greater overlap between photographs was required.

#### ii) 3D positioning of the common feature point relative to overlapping images

**Fig 2:**
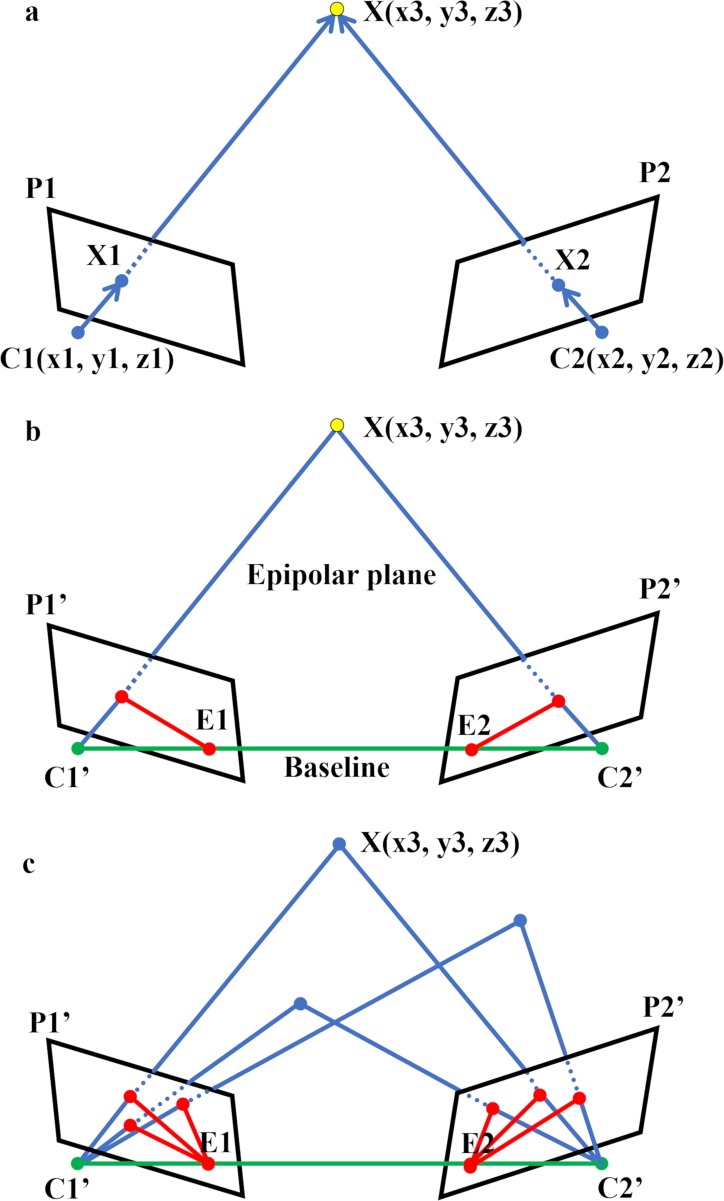
3D determination of the target position relative to cameras using epipolar geometry. **a** Conventional triangulation: The positions of two cameras C1(x1,y1,z1) and C2(x2,y2,z2) are known. Since the target X is projected on a X1, X2 on images P1, P2, the position X(x3,y3,z3) can be calculated as a point where a straight line connecting C1 and X1 overlaps with a straight line connecting C2 and X2. **b** Epipolar geometry: Because the positions of cameras were unknown in this study, the putative position of the target relative to the cameras was calculated as follows. The plane consisting of X and the positions of virtual cameras C1’ and C2’ is called an epipolar plane. The straight line connecting C1’ and C2’ is called a baseline (green line). The line where the epipolar plane intersects P1’, P2’ is called an epipolar line (red line), and the point where the baseline and the epipolar line intersect is called an epipole (E1, E2). **c** Positioning of X and cameras when the positions of the cameras are unknown: When there are multiple target points, the epipolar plane moves, but the baseline is fixed, so all epipolar lines go through the same epipole. This principle can be used to calculate the relative positions of the two cameras and the common targets in 3D space. C1,C2: camera positions. C1’,C2’: virtual camera positions. P1,P2: images taken by C1,C2. P1’, P2’: images taken by C1’,C2’. X: target point. X1,X2: X on P1,P2. E1,E2: epipole.

In conventional 3D modeling, the spatial relationship between the two camera positions, C1 and C2, and the target X is known (Fig 2A). Thus, the 3D position of any point on target X can be calculated by triangulation from the images obtained from C1 and C2. However, since the camera positions were not fixed in the method we performed, a calculation called "epipolar geometry" was used to estimate the positions of C1, C2 in 3D space relative to the target X (Fig 2B). The plane consisting of the target point and the positions of virtual cameras C1’, C2’ is called an epipolar plane, and the straight line connecting the cameras is called a baseline (Fig 2B). The line where the epipolar plane intersects the photographs P1’, P2’ taken by the cameras C1’, C2’ is called an epipolar line, and the point where the baseline and the epipolar line intersect is called an epipole (Fig 2B). When there are multiple common feature points, the epipolar planes shifts, but the baseline remains constant, so all epipolar lines go through the same epipole (Fig 2C). Using this property, the relative positional relationship of the cameras can be obtained, and we used this method to extrapolate the relative positions of the camera to the target for all photographs.

#### iii) 3D model reconstruction

Once the positional relationship of the cameras is obtained, the position of any point on a target can be calculated from two photographs ([14], Fig 2A). If similar calculations are done for multiple picture combinations, some errors will occur in the coordinates target points. Therefore, additional calculations are necessary to minimize these errors to develop an accurate 3D surface model. We used the Multi-View Stereo (MVS) algorithm [14] to perform these calculations.

### Actual processing

The above photogrammetry was performed using the ReCap Pro cloud service provided by Autodesk (San Rafael, CA, USA, https://www.autodesk.co.jp/products/recap/overview). Multiple 2D images (from 60 to 80 images per hemisphere) were uploaded. Reconstruction was performed on the cloud and took several hours.

## Results

**Fig 3:**
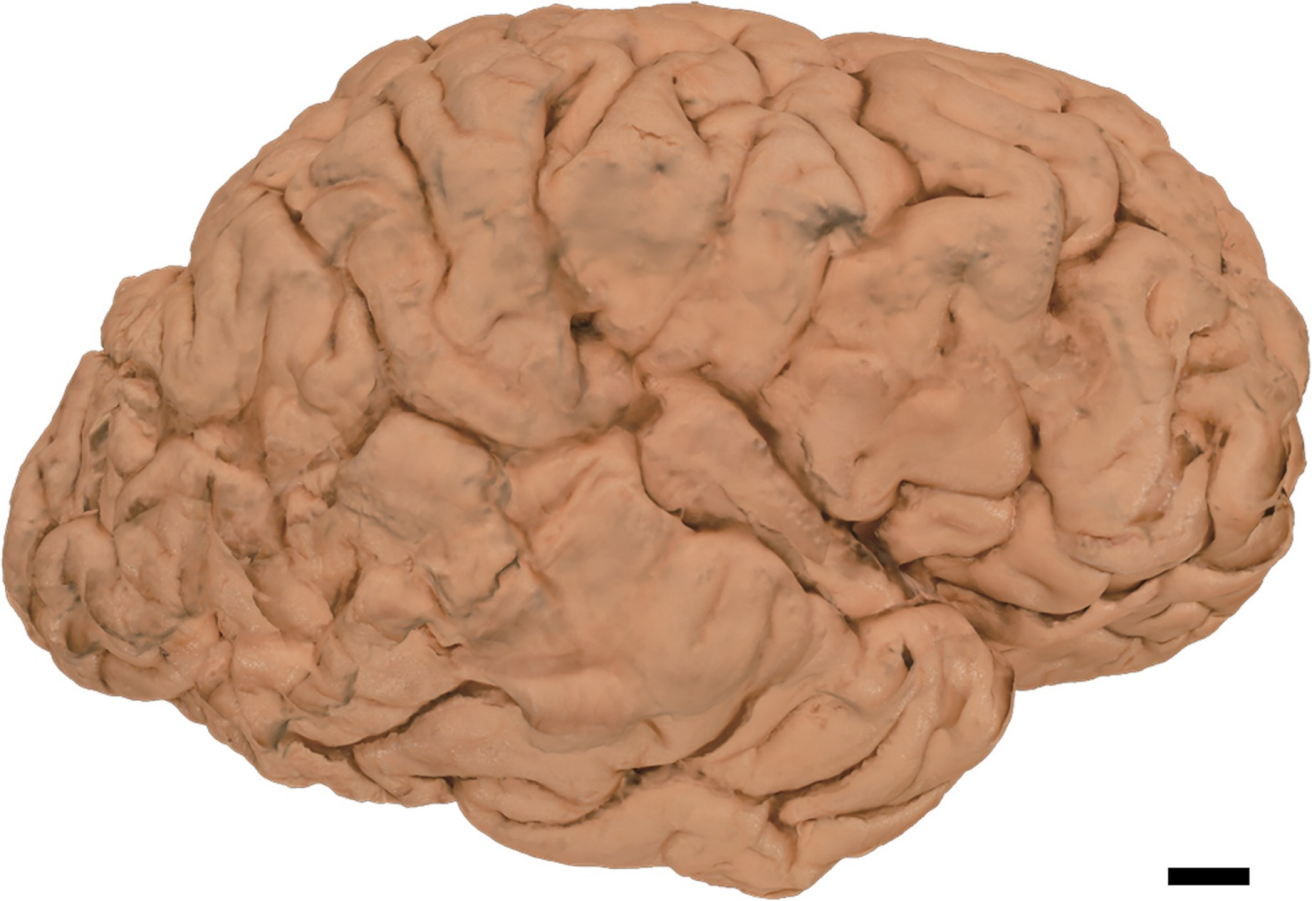
Case 1. Macroscopic photo of formalin-fixed human normal brain hemisphere with no neuropathological signs (right hemisphere). Scale bar = 1 cm.

**Fig 4:**
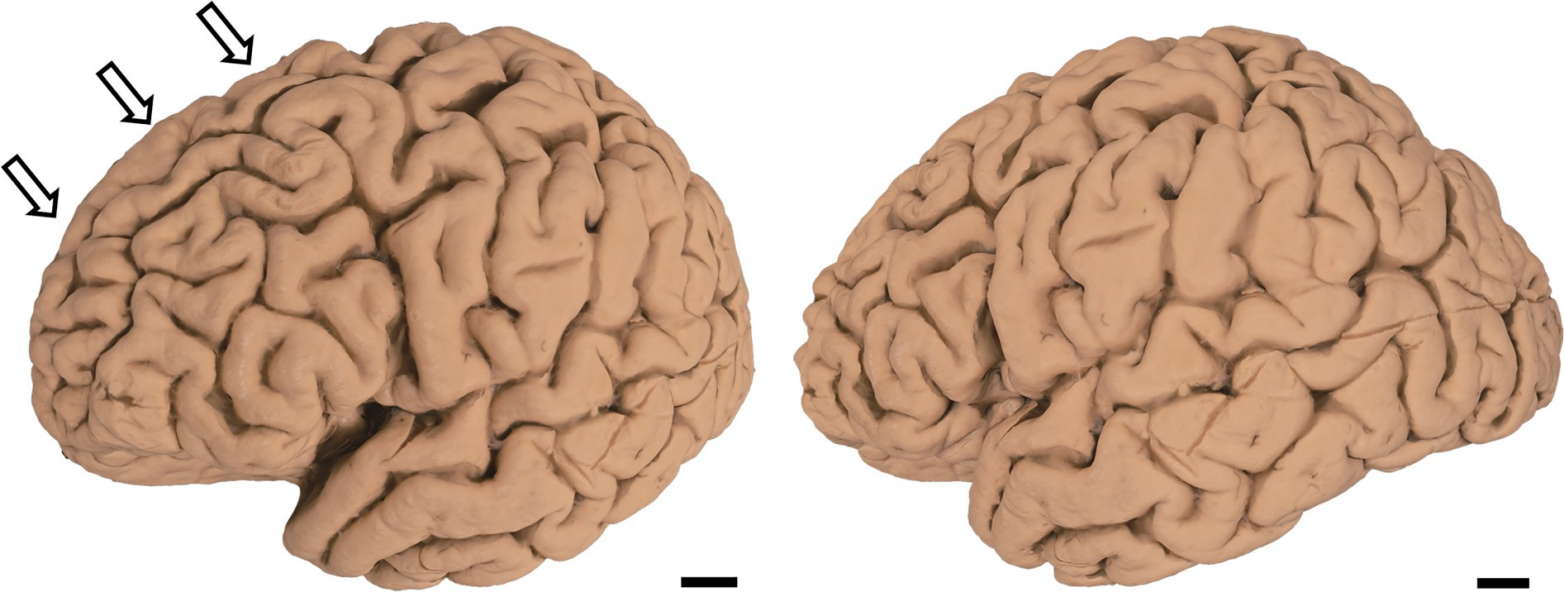
Case 2. Macroscopic photo of formalin-fixed human PSP brain hemisphere (left hemisphere): Mild atrophy is present in the frontal lobe (arrows). Scale bar = 1 cm.

**Fig 5:**
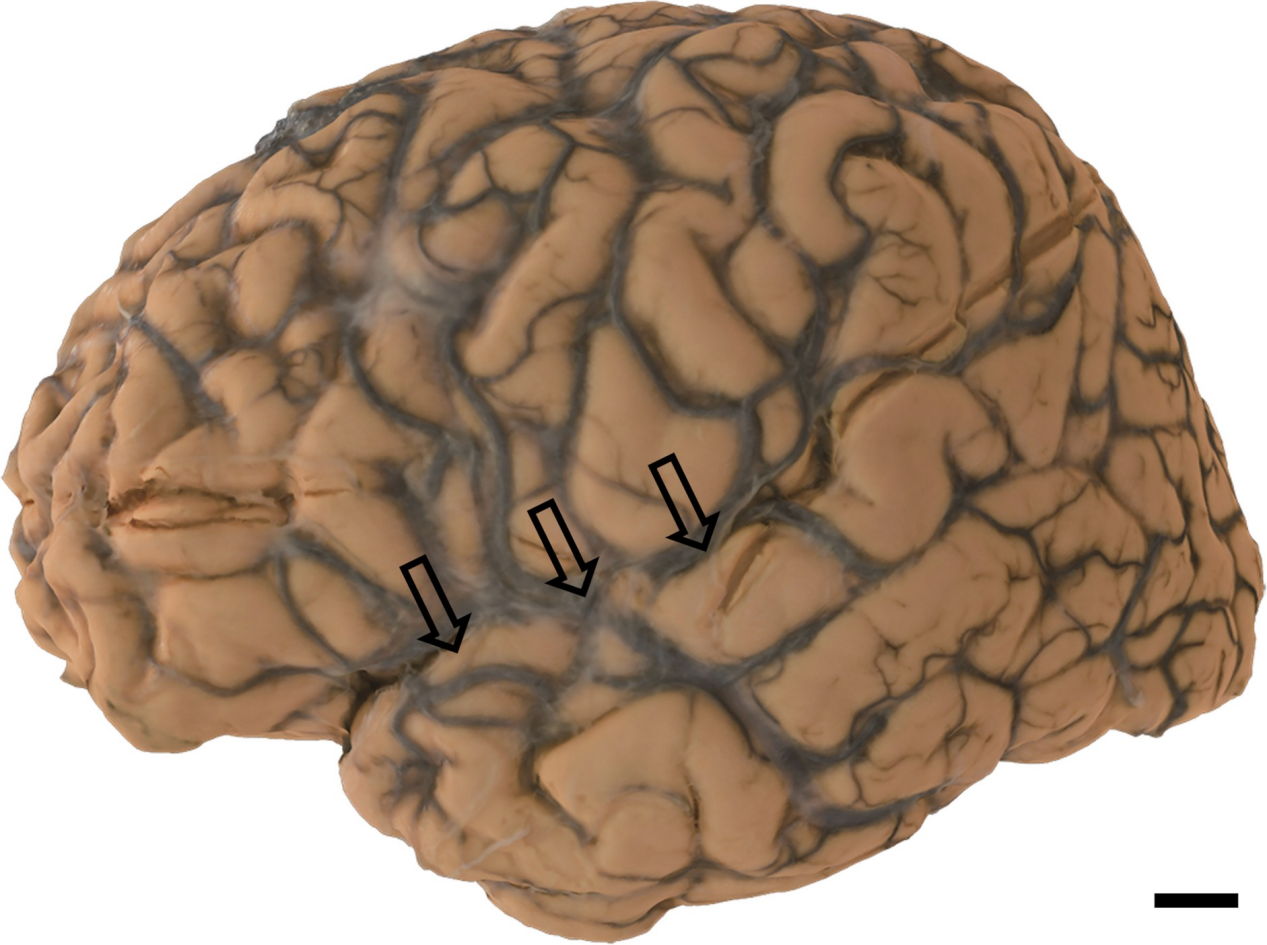
Case 3. Macroscopic photo of formalin-fixed human ALS brain hemisphere (left hemisphere): Subtle atrophy is seen in the superior temporal gyrus (arrows). Scale bar = 1 cm.

60 to 80 2D photographs were obtained by photographing cerebral hemisphere as described in the Materials and Methods section. Using the ReCap Pro cloud service, 3D surface models were reconstructed from these images (S1–S3 Movies, Figs 3–5). Reconstructed models can be observed from any direction at different magnifications and can be moved and rotated virtually. Movement and rotation greatly enhance 3D recognition of these models, even though they are observed on 2D displays (see Case1, S1 Movie, Fig 3 for the “normal” brain hemisphere). In PSP (Case 2), mild atrophy in the frontal lobe can be observed in a 2D photograph (Fig 4 arrows). This atrophy is more conspicuous on the 3D surface model (S2 Movie). In ALS, subtle atrophy in the superior temporal gyrus may be present in 2D photographs (Case 3, Fig 5 arrows), and again, this subtle atrophy is more readily recognizable in the 3D surface model (S3 Movie). Scale bars can be incorporated in each model. However, while scale bars can be labeled in individual 2D images or captured frames from our 3D models, they cannot yet be incorporated into movies or real time manipulations of our models. Thus, S1–S3 Movies do not include scale information.

## Discussion

We established a novel method of reconstructing 3D surface models of autopsied human brain hemispheres. This was achieved by synthesizing multiple 2D photographs into 3D models that can be viewed from any direction at different magnifications. In the PSP brain, the occipital lobe was relatively well preserved, while atrophy was evident in the frontal lobe. Frontal lobe atrophy in PSP is frequently observed when examining autopsied brains. However, this atrophy can be difficult to record using conventional 2D photographs. More restricted atrophy, such as in the superior temporal gyrus of an ALS brain is readily recognizable using this strategy. In ALS, accumulation of TAR-DNA binding protein 43 (TDP-43) is frequently observed in the superior temporal gyrus, an area preferentially affected in ALS [15]. In our 3D model, atrophy in the superior temporal gyrus is recognizable macroscopically, and may be associated with certain types of ALS. Thus, atrophy to this gyrus may suggest ALS even before confirmation through microscopic examination.

Our method allows construction of 3D surface models even without any knowledge, experience and skills in neuropathology. Thus, although macroscopic diagnoses are highly dependent on the knowledge, experience and skills of the examiner, using 3D surface models, diagnoses can be performed long after autopsies by experts that may not have been present during the autopsy or don’t have access to the actual brain. Furthermore, retrospective reviews of the data and comparisons between cases using 3D models may greatly enhance the reliability and objectivity of macroscopic findings of the human brain.

Although photogrammetry is used in various fields, there are few reports in the field of neuroscience [1, 2, 7, 8, 10, 11]. A recent report demonstrated 3D architecture of white matter fibers, manually dissected to demonstrate their 3D trajectories, in the human brain [1]. In this report, the camera was fixed while the target was placed on a turntable so that multiple photographs with different projections were obtained [1]. Because the distance between the camera and the brain was fixed, calculations based on the absolute distance for 3D reconstruction were simpler [1]. Since the brain surface on the turntable, opposite to the camera, is not accessible, the resultant 3D model represents only a part of the brain [1]. Our method, with the brain placed on transparent acrylic plate, allows the brain to be photographed from all directions to reconstruct the surface of the entire brain hemisphere. Because the distance and positional relationship between the camera and the brain was not fixed, calculations for 3D reconstruction were more complicated, and required sophisticated software.

The application of photogrammetry to neuroscience is not novel. More than 50 years ago, Mannen and colleagues successfully reconstructed a 3D model of Golgi-stained neurons, which provided a stereoscopic view of neuronal soma and neurites extending in 3D space of 250 μm-thick preparations [2]. To generate this model, the preparation was placed on a tilting stage so that 2 photographs, one tilted 12 degrees relative to the other were obtained [2]. Binocular comparison of the two photographs, allowed the contours of the neuron to be identified at different depths [2]. A similar map of the opposite side of the neuron was obtained by inverting the preparation, and both sets of data were combined to obtain the contour of the entire neuron in a 3D model [2]. Based on this model, it was possible to quantify the surface area and volume of neurons directly for the first time [2].

Although the 3D model of Mannen et al. was relatively accurate, its 3D-representation along Z axis was much more coarse than along the XY plane because they generated their model from only two photographs with a 12 degree difference [2]. We circumvented this lack of resolution along a particular axis by taking multiple photographs from different locations around the hemisphere. Our approach allows us to observe the brain extremely accurately from different directions without distortion.

Humans and other animals generate 3D representations of a target through 2D images projected on to the retinas of the right and left eyes. There are various cues for human to perceive depth [16, 17, 18]. For example, there are cues that can be used with monocular vision, such as relative size, perspective, shading, etc [16, 17]. In paintings, depth may be expressed by combining these cues. Another useful depth cue arises from binocular vision. The retinal images projected on the left and right eyes are slightly different, and depth is recognized in real time using these differences [16]. Photogrammetry uses these cues to create a 3D model as first reported by Mannen et al [2].

While our method generates 3D models, they are projected onto 2D displays. How are 3D surfaces perceived on 2D displays where binocular cues for depth are not available? 3D recognition is enhanced when models are rotated (S1 Movie). Motion parallax is the amount of relative retinal image shift that occurs when an image projected onto the retina moves [16]. For example, when an object fixed the line of sight becomes larger, you recognize that it is approaching. Even on 2D displays, motion parallax provides 3D imagery to brain hemispheres in rotation or motion. Our method makes it possible to see objects more stereoscopically by adding motion parallax to monocular cues.

Mapping microscopic findings to specific anatomical localizations in the brain is still a challenge in neuroscience. Currently, 2D photographs of external surfaces and of sliced brains combined with examiner’s impressions, are used to guide sampling for subsequent microscopic examination. However, this type of guidance requires skilled interpretation of macroscopic findings by experts. Even with skilled interpretation, anatomical localization of histological preparations is not precisely reproducible. Our 3D model may provide more precise coordination between histological samples and brain locations, although this coordination is limited to the surface area. Furthermore, our 3D model may be useful for quantifying the volume and surface area of human brain regions as has been initially achieved on reconstructed neurons by Mannen et al. [2]. Finally, our 3D model will provide an objective standard for macroscopic examination and recording of the brain surfaces, especially after a digital library of 3D surface models is expanded sufficiently to compare disease-specific changes.

There are at least two limitations to this study. One is that the brain surface that faces the acrylic plate is flattened when placed on it. A second is that our models cannot reconstruct the internal structures of the brain. However, as far as the areas visible from the surface is concerned, our models are superior to 2D photographs in accurately understanding how macroscopic findings are related to clinical imaging (CT, MRI and other functional imaging), clinical manifestations and sampled histological sections.

## Conclusion

We developed a novel method to reconstruct 3D surface models of autopsied human brains from multiple 2D photographs. Being able to manipulate and rotate models to view the brain surface from different positions and magnifications enhances the recognition of subtle macroscopic changes. Once a change is found, an image from a particular direction and magnification, which shows this change, can be recorded digitally, even though this image was not part of the original 2D photographs from which the model was constructed. The precise 3D localization of disease-associated lesions will be useful in providing connections between clinical manifestations, clinical imaging and histological findings.

## Supporting information

S1 Movie 3D surface model of a normal brain hemisphere. The brain can be observed from different directions and different magnifications. (MOV 3.25mb) Scale data is incorporated in each model, but were not incorporated into S1–S3 Movies.
(MOV)

S2 Movie 3D surface model of the hemisphere of the PSP brain. Mild frontal atrophy noted in conventional 2D photographs (Fig 4) is readily recognizable in the frontal lobe. (MOV 1.07mb)
(MOV)

S3 Movie 3D surface model of the hemisphere of the ALS brain. Subtle atrophy noted in conventional 2D photographs (Fig 5) is more easily recognizable in the superior temporal gyrus. (MOV 0.98mb)
(MOV)
